# Tenosynovial Giant Cell Tumors in the Hand and Foot

**DOI:** 10.7759/cureus.92785

**Published:** 2025-09-20

**Authors:** Nikolaos Stefanou, Sokratis Varitimidis, Elena Manuela Samaila, Athanasios Koutis, Theofilos Karachalios, Zoe Dailiana

**Affiliations:** 1 Department of Orthopaedic Surgery and Musculoskeletal Trauma, University Hospital of Larissa, Larissa, GRC; 2 Department of Orthopaedics and Trauma Surgery, University of Verona, Verona, ITA; 3 Department of Hand, Upper Extremity and Microsurgery, IASO Thessalias, Larissa, GRC

**Keywords:** foot, giant cell tumor, hand, surgical excision, synovial tumor, tendon sheath

## Abstract

Background: Giant cell tumor of the tendon sheath (GCTTS) is one of the most common benign, slow-growing soft tissue tumors of the hand and foot. This study aimed to evaluate the anatomic distribution of the GCTTS and correlate the surgical treatment with clinical outcomes and recurrence rates.

Methods: In this retrospective study, a total of 78 cases of GCTTS of the hand and foot operated from January 1, 2003 to December 31, 2020, with anatomopathological confirmation of diagnosis, were included. Patients were investigated with plain radiographs of the involved anatomic area and magnetic resonance imaging (MRI). All tumors were excised under regional anesthesia, tourniquet control, and magnifying loupes.

Results: The most frequent symptom at admission was a painless, mobile, and lobulated subcutaneous mass, which gradually increased in size. The average time period between the onset of the symptoms and the clinical evaluation was 5.6 months. The study contained 30 male and 48 female patients, and the average age was 46.31 years. The tumor was located on the upper extremity in the majority of cases (92.3%) with a 12:1 hand-to-foot proportion. Of the lesions, 22.23% were located in the thumb, 20.82% in the second digit, 16.75% in the third digit, 11.12% in the fourth digit, and 20.82% in the fifth digit. Around 50% of foot lesions were located in the hallux. According to the pathology reports, the mean dimension of hand tumors was 1.2 cm at the greatest axis, and of the foot tumors, it was 3.3 cm. There was no significant correlation between the anatomic distribution of GCTTS, gender, age, size of the tumor, and recurrence rates. Recurrence was noted in five cases (6.4%).

Conclusion: Accurate imaging of GCTTS with MRI and plain radiographs, followed by radical excision of the lesion, is fundamental for minimizing recurrence potential, which should be underlined to the patient.

## Introduction

Giant cell tumor of the tendon sheath (GCTTS) is a benign, slow-growing, usually painless, mesenchymal soft tissue tumor originating from the tendinous, peritendinous, or articular synovial membrane of the extremities. It was first described by Chassaignac in 1852, and since then, several terms have been used to describe this lesion, including pigmented villonodular tenosynovitis (diffuse type considered a synonym due to the histological similarities), localized nodular synovitis, fibrous xanthoma, xanthogranuloma, fibroxanthoma, benign synovioma, giant-cell fibrous hemangioma, and xanthomatous histiocytic granuloma [1,2]. Even though the cytogenetic and molecular features of GCTTS are still not clarified, the most accepted causative theory admits a reactive or regenerative hyperplasia associated with inflammation characterized by aggregation of fibroblasts, macrophages, foam cells, multinucleated giant cells, and hemosiderin deposits [3,4].

GCTTS is the second most common tumor of the hand after ganglion cysts, affecting mainly women (slight predominance of 3:2 to male individuals) between the ages of 30 and 50 years, with a prevalence of 1 in 800,000 [5-7]. It generally occurs on the volar aspect of the radial three digits and the distal interphalangeal joint region (approximately 80% of the cases), but also in other areas of the body, such as the foot (3%), wrist, elbow, ankle, knee, and hip. The biological behavior of GCTTS can be classified as localized (less aggressive, develops around the tendon sheaths of the hand and foot) or diffuse (more aggressive, develops intra-articularly in major joints) based on growth pattern [8,9].

The lesion does not transilluminate, plain radiographs may demonstrate a soft tissue mass or pressure erosion of underlying bone, and magnetic resonance imaging (MRI) reveals decreased signal intensity on T1- and T2-weighted images, aiding in both diagnosis and preoperative planning [7,10]. The current standard treatment of choice for GCTTS is marginal excision, with or without adjuvant radiotherapy, but despite its benign character, local recurrence has been reported in up to 50% of patients [10,11]. Several risk factors have been associated with recurrence rates, including incomplete excision, radiological bone erosion, size greater than 2 cm, location at the interphalangeal joint of the thumb and distal interphalangeal joints, presence of degenerative joint disease, tumors with increased mitotic activity, tumors that are nm23-negative, tendon or neurovascular involvement, and type 2 lesions according to the Al-Qattan classification [3,11-13]. Malignant form of GCTTS is extremely rare in the literature but is characterized by a high incidence of lymph node metastasis (41%) and a high mortality rate (30%) [14].

The aim of this retrospective study was to evaluate the anatomic distribution of the GCTTS on the hand and foot, to correlate the surgical treatment with clinical outcomes and recurrence rates, and to identify the relationship of the preoperative imaging diagnosis with the pathologic findings.

## Materials and methods

This retrospective study was conducted in the Department of Orthopaedic Surgery and Musculoskeletal Trauma of the University Hospital of Larissa and the Hand, Upper Extremity and Microsurgery Department of IASO Thessalias. A total of 78 cases of GCTTS operated on by the same group of three experienced surgeons, sharing the same surgical principles, between January 1, 2003 and December 31, 2020 were included in this study. IRB/Ethical Committee of University Hospital of Larissa issued approval, which was registered with the number 11165. The inclusion criteria consisted of hand and foot tumor surgeries performed in the two institutions during the predefined period, presenting diagnostic confirmation of GCTTS by anatomopathological examination. Patients whose tumors were not confirmed as GCTTS histologically were excluded from the study.

**Figure 1:**
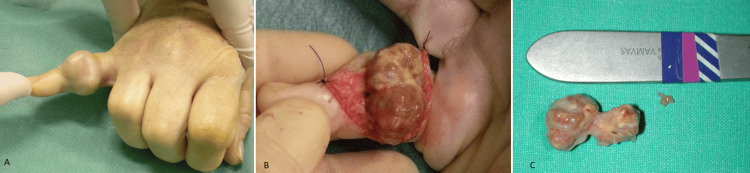
Intraoperative view of GCTTS of the hand (A) Painless, mobile, nodular mass on the little finger of the right hand, affecting the dorsal, radial, and volar aspects of the base of the middle phalanx. (B) Intraoperative view depicting the macroscopic appearance of GCTTS and the involvement of the neurovascular bundle. (C) Type Ic lesion (Al-Qattan classification): Multilobulated lesion surrounded by a common pseudocapsule. GCTTS, Giant cell tumor of the tendon sheath

**Figure 2:**
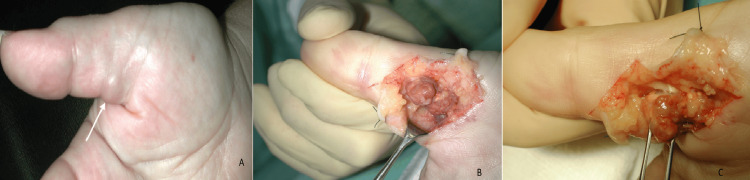
Giant cell tumor of the thumb (A) Giant cell tumor of the tendon sheath in the volar aspect of the base of the proximal phalanx of the right thumb (white arrow). (B) Intraoperative image of the tumor with the brown color and lobular appearance during the first stage of its radical surgical resection. (C) Dissection of the tumor with part of the tendon sheath (the flexor pollicis tendon is marked with the white arrow).

**Figure 3:**
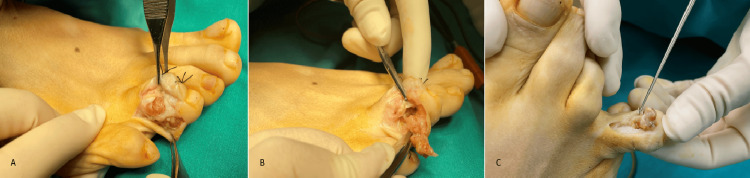
Intraoperative view of GCTTS of the foot (A) Exposure of the mass and partial dissection by a lateral incision over the tumor. (B) The GCTTS had destroyed the cortex and invaded the shaft of the middle phalanx of the fourth digit of the foot. (C) The tumor almost completely encircles the phalanx, and a double incision was used for the radical excision. GCCTTS, Giant cell tumor of the tendon sheath

**Figure 4:**
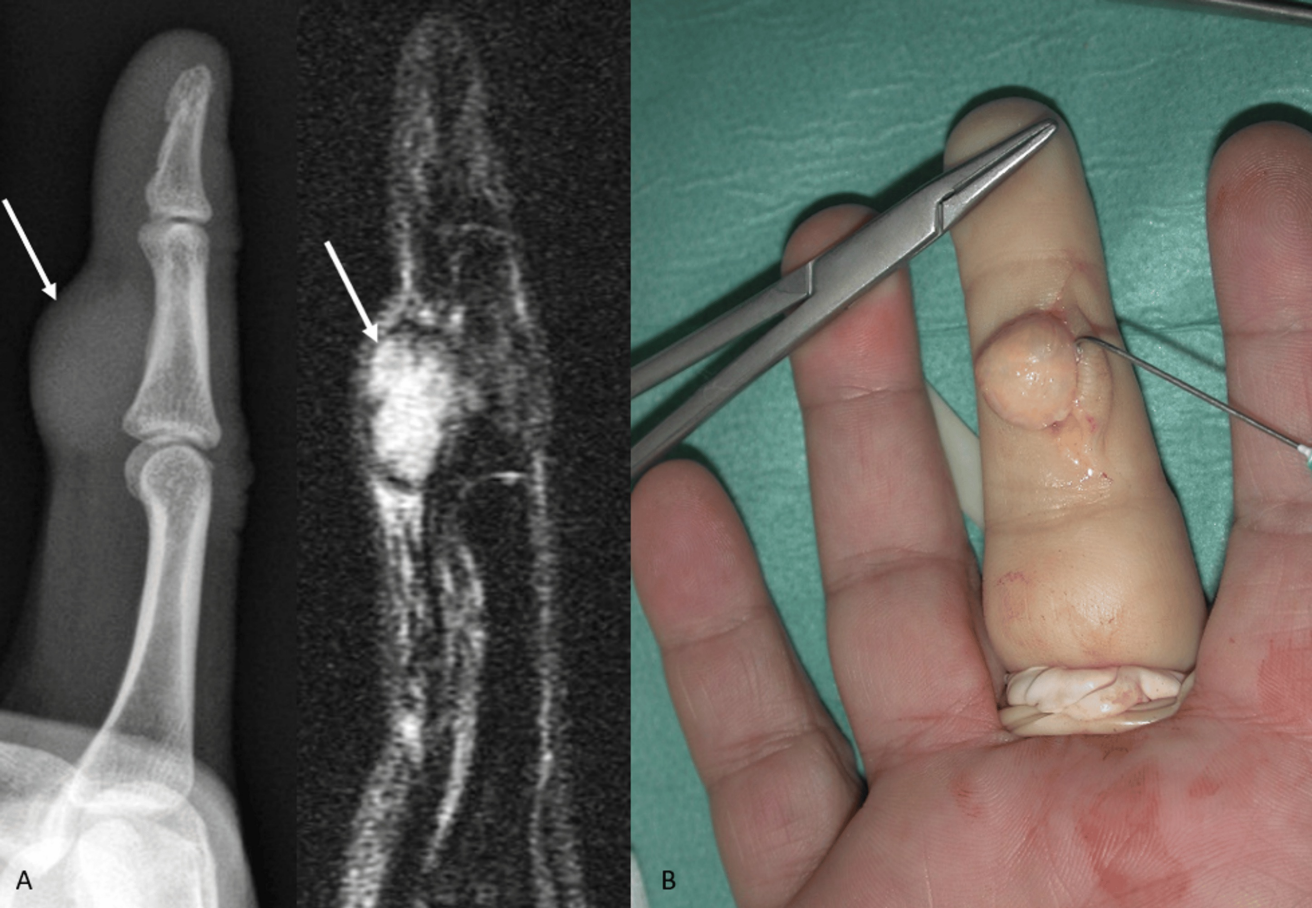
Imaging and intraoperative view of a middle finger GCTTS (A) Lateral radiograph depicting the soft tissue shadow and scalloping of the middle phalanx shaft of the middle finger and corresponding MRI image that clearly defines the dimensions of the mass and its relation to neighboring structures (marked with the white arrows). (Β) Intraoperative view of a type Ia mass (Al-Qattan classification) without satellite lesions and with clear boundaries of the sample.

The most frequent symptom at admission was a painless, mobile, and lobulated subcutaneous mass of the hand or foot, which gradually increased in size (Figures 1-3). The average time period between the onset of the symptoms and the clinical evaluation in the outpatient clinic was 5.61 months (range: 2 months-1.3 years). Patients were investigated with plain radiographs of the involved anatomic area and MRI (Figure 4). All tumors were excised under regional anesthesia, tourniquet control, and magnifying loupes, with palmar, dorsal, plantar, or mid-lateral approach. After microsurgical dissection of the neurovascular bundle, the marginal excision of the tumor was accompanied by partial excision of the tendon sheath and redundant skin when necessary. After the removal of the tumor, a thorough check was made for residual or satellite lesions, and tissues that were considered suspicious were removed with healthy margins around them. Τhe patients followed a rehabilitation program that included both passive and active movements from the first postoperative day, and the follow-up included initial evaluation at 2-3 weeks and regular control at 6 and 12 weeks, and at 6 and 12 months.

Statistical analysis

Statistical analysis was conducted using SPSS software version 26 for Windows (IBM Corp., Armonk, NY) to analyze the study data. All continuous variables followed a normal distribution according to Shapiro-Wilk and Kolmogorov-Smirnov normality tests and were presented as mean values. To compare categorical variables and recurrence rates, chi-square tests were used with a p-value threshold of 0.05. Chi-square tests and linear regression analysis were also used to evaluate the correlation between the appearance and recurrence of a giant cell tumor with the remaining categorical and continuous variables.

## Results

The patients were female in the majority (48/78, 61.53%), and the average age was 46.31 years (range: 6-80 years). The mean length of follow-up was 4.52 years (2-9 years). The tumor was located in the hand in the vast majority of cases (72/78, 92.30%), with a 12:1 hand-to-foot proportion. The digital distribution was similar for the hand fingers (except the ring finger), while for the foot toes, most of the tumors were located in the hallux. In more detail, 16 lesions were located in the thumb (22.23%), 15 in the index finger (20.82%), 12 in the middle finger (16.75%), 8 in the ring finger (11.12%), and 15 in the little finger (20.82%). The remaining six tumors of the hand (8.26%) were located in the palm (five on the volar and one on the dorsal side). The digital tumors were located, in the majority of cases, at the lateral side of the finger, raising the neurovascular bundle, with dorsal or palmar extension (Figures 1, 2, 4). Of the six lesions of the foot (7.69%), three were located in the hallux and one each on the third, fourth, and fifth toes. Of the 72 hand GCTTS, 54 involved the right hand (75%) and 18 the left hand (25%), while four of the six GCTTS of the foot involved the right (66.66%) and two the left (33.33%) foot (Tables 1, 2).

**Table 1:** Anatomical distribution of GCTTS in the hand and foot GCTTS, Giant cell tumor of the tendon sheath

Limb affected	Thumb	Index finger	Middle finger	Ring finger	Little finger	Hand	Total
Right hand	11	12	8	6	12	5	54
Left hand	5	3	4	2	3	1	18
Total	16	15	12	8	15	6	72

Limb affected	Hallux	2^nd^ toe	3^rd^ toe	4^th^ toe	5^th^ toe	Foot	Total
Right foot	2	-	1	1	-	-	4
Left foot	1	-		-	1	-	2
Total	3	-	1	1	1		6

**Table 2:** Recurrence rates according to anatomic distribution of the lesions

Recurrence	Thumb	Index finger	Middle finger	Ring finger	Little finger	Foot	p-value
Yes	-	1	1	-	2	1	0.82
No	16	14	11	8	13	5	

The tumors were all extra-articular and well-circumscribed, encapsulated masses of varying size. Based on clinical and imaging evaluation (plain radiographs and MRI), the tumor was preoperatively diagnosed as GCTTS in 72 of 78 cases (92.3%), partially or completely enveloping a tendon, well marginated, with small scattered foci and capsules of hypointensity on T1-weighted images and T2-weighted images, and anatomic localization. According to the pathology reports that confirmed the diagnosis of GCTTS, the mean dimension of hand tumors at the greatest axis was 1.2 cm and of the foot tumors was 3.3 cm.

Recurrence was noted in five (four hands and one foot) cases (6.41%), and all of them except one were successfully managed with a second wider radical excision. One patient, initially operated on in another center by a non-hand specialist, had multiple recurrences, necessitating two revision procedures. Radiotherapy was not applied for recurrent tumors, and no amputation was needed. Bony involvement was observed in 8 out of 78 (10.25%) patients, and thorough curettage of the cortical shell was performed in order to prevent recurrence. There was no significant correlation between recurrence, anatomic distribution of GCTTS, bony involvement, gender, age (p-value = 0.42), and the size of the tumor (p-value = 0.56) (Tables 3, 4). Transient sensory impairment was recorded in three (3.84%) cases. In one case, with a circumferential lesion, a transient vascular spasm was recorded. Skin necrosis or infection was not observed in any case of the present series, and no tumor was characterized as malignant by the pathologist. Four patients presented with stiffness but regained a satisfactory and functional range of motion in an average period of 4.23 months postoperatively.

**Table 3:** Recurrence rates regarding bony involvement

Recurrence	Bony involvement (+)	Bony involvement (-)	p-value
Yes	1	4	0.45
No	7	66	

**Table 4:** Recurrence rates according to gender

Recurrence	Male	Female	p-value
Yes	1	4	0.38
No	29	44	

## Discussion

GCCTS is mainly located adjacent to the tendon sheath of the hand and foot. Adequate surgical resection, when feasible, remains the treatment of choice even though, since 2019, pharmaceutical treatment options have been introduced for adult patients with symptomatic GCCTS associated with severe morbidity or functional limitations that were not amenable to surgical treatment [3,7,15-17].

GCCTS is more common between the ages of 30 and 50 years, with a female predominance [5,7,18]. In accordance with most of the studies, our sample’s average age was 46.3 years, with a female-to-male ratio of 1.6, but interestingly, 24 out of 78 (30.8%) patients were aged outside the usual age range of 30-50 years. Although common in the adult population, the lesions are quite uncommon in children, and even rarer when they affect the feet [19]. In the current study, two patients (2.6%) were aged less than 18 years. Our findings suggest that the expected age group of our patients for this pathology should not be a determining factor that will direct our diagnostic approach.

According to the literature, the typical detection site of the tumor is on the palmar surface of the three radial fingers of the hand. On the foot, the GCTTS develops most commonly in the forefoot, especially on the dorsal surface [6,8,20,21]. In the present study, most of the hand tumors were located on the volar aspect of the palm and on the lateral side of the fingers with palmar or dorsal extension, with similar distribution in all fingers except the ring finger, while on the foot, all lesions were located in the lateral or plantar aspect of the fingers. We could assume that this is related to the distribution of greater mechanical loads in the palmar or plantar anatomic regions, and possibly due to repeated microinjuries in daily activities (walking cycle, precision pinch, oppositional pinch, key pinch, hook grip, or power grasp).

Although plain radiographs may reveal cortical scalloping, soft tissue swelling, cysts, or intraosseous involvement, MRI is the optimal modality for preoperative differential diagnosis, assessment of tumor size, and outspread to the surrounding tissues [18,22]. Localized GCTTS typically exhibits small, scattered foci of low signal on T1 and T2 images due to the presence of hemosiderin. The lesion may also be characterized by a low signal intensity capsule because of fibrosis or hemosiderin deposition [18]. These findings contribute particularly to differentiate GCTTS from other solid soft tissue tumors of the extremities, such as ganglion, nerve sheath tumor, hemangioma, neurofibroma, or synovial sarcoma. In the present study, the combination of clinical examination, plain radiographs, and MRI set the diagnosis of GCTTS in 92.3% of the cases. We believe that accurate preoperative assessment of the mass should guide the patient to a specialist surgeon, resulting in better management with decreased complication and recurrence rate.

Local recurrence has been reported in up to 50% of the patients, and sometimes multiple recurrences were recorded in the same patient [3,8,11-13]. In the current study, the recurrence rate was 6.4%, and none of the risk factors reported in the literature was correlated to this outcome, with the exception of inadequate surgical excision of all involved tissues. Οf the five cases with recurrence, one was operated on in another hospital by a non-specialist, while in three cases, the extent of the lesion obviously prevented radical excision. Thus, the authors agree with Palmerini et al., who stated that complete resection remains the mainstay of treatment, but the surgeon should always have in mind the equilibrium between the quality of surgical margins and functional or sensory preservation [23].

## Conclusions

GCCTS is a benign tumor that rarely causes serious functional impairment to the patient and is characterized by a low incidence rate in the population. On the other hand, it is one of the most frequently occurring masses of the extremities, representing a treatment challenge for every orthopedic surgeon due to the close anatomic relation to the neurovascular bundle and the well-documented high recurrence potential, which should be underlined to the patient. Based on the data obtained from our study, we determined that accurate preoperative imaging diagnosis with plain radiographs and MRI, followed by radical excision of the lesion by an experienced hand or foot surgeon, using magnifying loupes, is a fundamental approach for minimizing the complications and recurrence. We also concluded that there is no significant correlation between recurrence rate, anatomic distribution of GCTTS, bony involvement, gender, age, and the size of the tumor. The limitations of the study, such as the lack of molecular analysis and the need to correlate surgical and pharmaceutical management with clinical scores, should form the basis for designing future studies. 
